# Determining a role for Patient and Public Involvement and Engagement (PPIE) in genomic data governance for cancer care

**DOI:** 10.1038/s41431-025-01866-1

**Published:** 2025-05-23

**Authors:** Katherine Sahan, Lesley Turner, Nina Hallowell, Michael Parker, Anneke Lucassen

**Affiliations:** 1 Ethox Centre, Oxford Population Health, University of Oxford, Big Data Institute, Old Road Campus, Roosevelt Drive, Oxford, UK; 2 Patient Reference Panel, Cangene Canvar, London, UK; 3 Clinical Ethics, Law, and Society (CELS) Oxford, Nuffield Dept of Medicine, University of Oxford, Oxford, UK; 4 Centre for Personalised Medicine, University of Oxford, Oxford, UK; 5 Centre for Human Genetics, Oxford, UK

**Keywords:** Ethics, Cancer genomics

## Abstract

Comprehensive collections of cancer data, including genomic data, are needed to improve cancer risk prediction and treatments. A recent government review, *Better, Broader, Safer: Using health data for research and analysis*, has argued for high-quality Patient and Public Involvement and Engagement (PPIE) for ethical data use. In this paper we determine a role and justification for PPIE to govern uses of genomic data in fields like cancer. First, we analyse two public attitudes studies about the role of PPIE in genomics governance. Second, we characterise two ethically-significant features of the context of governing genomic data: 1) data aggregation leading to novel group formation, and 2) the hybrid territory of genomic cancer data uses. Thirdly, we bring together these aspects to describe a fully determined role for PPIE within an approach to governing cancer genomic data, which is tailored to major areas of ethical consideration. Our account is a novel interpretation of what PPIE is for in governance, how it may foster public support and how its success in so doing depends on it being tailored to context.

## Introduction

Approximately 1 in every 2 people in the UK will develop cancer in their lifetime [1]. Comprehensive collections of data about cancer patients enable understanding of the causes, prevention and treatment of cancer. These increasingly include—or link to—genomic data [2, 3]. As well as research, collections may be used for evaluating clinical performance, audit, and education [4]. Genomic data within collections can greatly assist understanding of cancer predisposition, progression and outcomes [5]. Using this data ethically is important for continued public trust and support.

Patient and Public Involvement and Engagement (PPIE) is increasingly part of discussions about public trust and data ethics^1^. For example, Goldacre and Morley’s recent review of using health data for research and analysis has recommended high-quality PPIE for achieving ‘productive and ethical’ data use [6]. Others have associated PPIE with what will secure public interests, trust or support [6, 10–16]. Still others have emphasized an important role for PPIE within research governance [17, 18], saying that ‘[p]ublic involvement in research governance can help research be more transparent and gain public trust.’ [18]. However, it is important to determine how precisely PPIE might function justifiably in particular governance contexts. In this paper, we seek to determine a role for PPIE in the context of governing genomic cancer data uses which is worthy of public support.

### Why focus on public support for cancer genomics?

The field of genomic medicine promises substantial future impacts in healthcare outcomes [12] and risk prediction [19]. In this paper, we focus on cancer genomics because of cancer’s high disease burden and public visibility, and concur with others that public opinion about genomic medicine might depend on its successes in improving cancer outcomes [20, 21]. It is also the case that cancer (and rare disease) has been a priority focus for major genomic medicine initiatives to date [3, 22–25]. These initiatives have seen cancer genomic data collections grow and be called upon to perform increasingly more sophisticated analyses, including long-read sequencing and multi-modal data analysis [23, 26], magnifying the scale and complexity of data analysis and linkage. Together this makes cancer genomics a priority area for developing data governance arrangements that are worthy of public support. While our focus here is on cancer genomics, the arguments we develop will have a wider applicability for genomic data governance as a whole.

To determine an appropriate PPIE role, we first identify the scope of such a role (subsection “What ought to be the role of PPIE in the governance of genomics?” of section “Methods”), then consider how to arrange governance appropriately (subsection “Arranging governance” of section “Methods”). This involves identifying and analysing ethically significant features when using genomic data for research and other activities in fields like cancer. Lastly, we describe a fully determined role for PPIE responsive to ethically significant features of the governance context.

## Methods

### What ought to be the role of PPIE in the governance of genomics?

We begin with an analysis of two recent studies about publics and (genomic) data governance. We go on to situate the role of PPIE within a governance approach which is sensitive to key ethical challenges of genomic data use in fields such as cancer (“Arranging governance” of section “Methods”).

#### Study 1: The Ipsos Mori dialogue

The Ipsos Mori dialogue was commissioned in 2019 by Genomics England [14]^2^. The study was a response to a 2016 UK Chief Medical Officer (CMO) report which called for a rethink of the ‘social contract’^3^ in healthcare in light of genomic medicine [27].

The study recommended a ‘lay expert’ panel to help prevent negative social outcomes from uses of genomic data such as a ‘stratified society which disenfranchises vulnerable members’ p37 [14]:

Ultimately, the public want policy makers to design a system that prevents these things [negative social outcomes] from happening in practice. Rational ignorance means participants are willing to defer its design to the NHS and experts with no vested interests (such as a panel of “lay-experts” e.g. 100,000 Genomes Project Participant Panel) *ibid*

The concept of ‘rational ignorance’ used here refers to the idea that participants may sometimes judge it reasonable to remain uninformed about the precise details of genomic data governance whilst nonetheless being content to participate in the research it governs. This gives scope to involve publics in a genomic data governance system but does not mandate their involvement [14]. Importantly, governance should be designed so that it supports and protects the interests of marginalised and underserved sections of society.

#### Study 2: National Data Guardian (NDG) Dialogue

The NDG Dialogue was conducted by Hopkins Van Mil and was commissioned in 2020 by the National Data Guardian for Health and Social Care (NDG) and by Understanding Patient Data [10]. It examines ‘how people assess public benefit in the use of health and adult social care data for purposes beyond individual care’ p5. The report recommends that authentic public engagement is a prerequisite for public benefit, warning that:

‘Public benefit is undermined if authentic public engagement is not integrated into data assessment. This requires engaging people from a cross-section of society in data assessment processes.’ p55.

Data assessment processes are not defined in the study, but the implication is that they include governance or strategic decision-making processes to judge whether data uses are publicly beneficial. Public engagement could include ‘a data assessment jury to be drawn on for complex ‘edge’ assessment cases with, for example an ethical dimension…’p55.

#### Analysis of these studies

What can be learned from these cases? Both studies support a role for PPIE in governance on the grounds of reduced inequalities in delivery of genomic medicine, and fostering well-founded public support (either through successful social contracting, or through robustly assessing public benefit). The NDG study emphasises PPIE should be authentic and non-tokenistic. Notwithstanding these areas of broad agreement, the two studies also have some unclear views about the role of PPIE in governance. We discuss these below to make progress on determining its role.

##### PPIE in relation to wider publics or society

The Ipsos study uses the concept of rational ignorance (RI) to explain why wider publics may reasonably choose to remain uninformed about the details of genomic data governance, deferring on these matters to narrower groups such as lay expert panels (p37 footnote^4^) [14]. However, care should be taken not to over-interpret this descriptive account of public engagement with genomics towards normative conclusions. Firstly, too much focus on wider publics choosing to remain uninformed will start to look like a reason not to do PPIE, disenfranchising publics or groups who already struggle to engage with genomics or access genomic services. Secondly, a focus on RI runs counter to a social contracting principle, also from the Ipsos study, that publics should ‘understand and appreciate the value of the care given’ in return for using genomic medicine services (p11) [14]. The principle suggests successful social contracting depends on publics making effort to understand aspects of genomics relevant to their care. These could include both what governance of genomic data entails (and why good data governance is important for their care) and complexities within care delivery itself e.g. that genomic results are far less deterministic than publics perceive and often have uncertain clinical significance. In light of this, one important role for PPIE within governance could be to judge what constitutes relevant and sufficient information for wider publics about how genomic data is governed and used in care. This would both be a way to represent the interests of wider publics within the governance process and help uphold social contracting (and public support).

##### PPIE constitution and procedural approach

How should PPIE be arranged within governance? The NDG study advocates PPIE, which engages a cross-section of society, suggesting convening ad hoc data assessment juries in cases with ‘an ethical dimension’ ([10] p.5). As well as the benefit of cross-sectional representation, such jury models are claimed as beneficial because they help publics think critically and give reasons for their views ([28, 29], p110). Finally, convening PPIE as a jury ad hoc might make governance more proportionate [6].

However, convening juries only in certain cases means PPIE is not integral to the governance process. This may be disadvantageous if we think PPIE should decide whether a planned data use requires ethics review, as well as contributing to the review itself. Secondly, developing and improving governance processes could be more complex without the institutional knowledge or memory of a regular or ‘standing’ PPIE input. This suggests that PPIE input should be regular not ad hoc.

##### Lay (PPIE) vested interests

It is problematic to suggest—as the Ipsos study appears to—that lay experts on a governance panel should not have vested interests. Like other experts, lay experts frequently have a particular interest in and experience of a disease area, joining governance or funding panels because of this. Rather than objecting to vested interests, it seems important that all governance panel members, lay or otherwise, openly acknowledge potential conflicts of interest and this is managed within governance processes. For example, composition of PPIE panels could be arranged to reflect broadly different attitudes among publics and patients to governing genomic cancer data collections. Patients are said to bring the value of their experience to PPIE activities [30]. Those patients with rare or complex diagnoses or care journeys may be more likely to support data uses, more excited about scientific discovery from these uses, and more minded to reduce barriers to use. In contrast, publics are said to be more ‘distinterested’, socially accountable and hold ‘public or common views rather than special expertise’ [30]. Publics might thus exercise more caution about uses, especially given a backdrop of prior data scandals [15] and discussions around harms from unethical personal data use [31].

### Arranging governance

It is important for PPIE to be tailored to the context in which it operates. In this section, we discuss two particularly significant features for how to arrange governance of genomic data usage appropriately in fields like cancer: (1) data aggregation leading to novel group formation; (2) the hybrid (research-clinical) territory often inhabited by genomic data uses. In doing this our aim is both to highlight ethical challenges particular to these kinds of data uses, and to make consideration of those challenges core to a PPIE panel’s role.

#### Data aggregation leading to novel group formation

The first ethical feature of interest arises out of the fact that certain uses of genomic cancer data will lead to new groups being characterised based on shared genetic characteristics. There are complexities and features of such aggregation to which governance should be sensitive. For example, the possibility that such groups could suffer disadvantage, discrimination (e.g. where large genomic data aggregates ‘reveal health patterns of a certain sub-group’ or perpetuate ‘strong racial biases’) or ‘dignitary harm’ p6 [32]. Such harms could be compounded if groups are already disadvantaged for some other reason e.g. racism. These harms are not uniquely associated with using cancer genomic data. Even so, the stigma of having cancer might be higher or lead to more harms e.g. higher insurance premiums.

Caution about novel group formation relates to a wider discourse about ethical challenges of diversifying genomic data [33]. In particular, there are profound complexities in the diversification project, namely attempts to identify and better represent underrepresented groups in genomics and so reduce health inequities. It is possible for diversification attempts to compound biases and assumptions arising from imposed social or political constructs such as race [34]. Some scholars have called for more nuanced approaches to analysis methods like genetic ancestry in genomic analysis, such as not imposing labels or categories on what has been termed the ‘continuous, category-free nature of genetic variation’ [35]. Others, while sharing the caution around such biases and assumptions also describe how genetic knowledge might help support the rights of (disadvantaged) ‘genetic citizens’^5^, and provide ‘leverage for activism and policy initiatives’ to address social and environmental determinants of health inequalities p.39 [34].

PPIE can be used to assess whether proposed data groupings might discriminate, cause dignitary harm or perpetuate inequality, drawing on lived experience of related cancers or the cancer susceptibility genes under study. As part of this, a PPIE role in governance could be to connect and consult with wider publics or communities implicated by the proposed uses.

#### Hybrid territory inhabited by genomic cancer data uses

The second feature of interest is whether genomic cancer data use needs to be understood as a ‘hybrid’ research-clinical activity [36, 37] for the purpose of arranging and conducting governance.

Data uses for cancer care rely on a hybrid combination of clinical and research activities. Data interpretation necessitates research input and research requires linked clinical details to make useful inferences. Machine learning (ML) is often used to combine and analyse heterogeneous data at scale and is commonly used in fields like cancer genomic data science to develop computational tools for gene detection and variation [38–40]. When certain types of ML such as adaptive ML are used, data uses also function to help the ML models continuously learn, and may be generating generalizable ‘research’ results for future, other patients [41].

This hybrid activity sits against a backdrop of historically distinct governance mechanisms for clinical practice and research. This is partly due to distinct normative commitments, centrally the pursuit of patient benefit (in clinical practice) versus developing generalizable knowledge (in research). Thus, on the face of it, it seems hard to reconcile such opposing commitments within genomic data governance for cancer care without risking unacceptable compromises on patient benefit and care quality.

One approach to arranging governance would argue that the activities (research and clinical) are so hybridised that we cannot or should not disentangle research and care aspects nor apply separate governance analyses/standards. This would be a ‘hybrid’ account of governance. A second approach would argue that irrespective of this hybridisation, use cases should be presented so as to separate out the research- versus clinical-ethics considerations, facilitating separate ethical analyses, even if occuring within a single governance process. We will call this the ‘separationist’ account.

##### The hybrid account

Developing this kind of governance account entails re-thinking how we should govern uses productively but with awareness of conventional distinctions. The concept of a Learning Health System (LHS) is helpful in this regard [37, 42]. For example, Faden et al. argue for a more ‘inclusivist’ approach to governance comprising clinical activities, research activities, and presumably work which classes as ‘other’ e.g. data driven work [43]. The challenges of this approach are how to tailor governance to a more diverse set of uses, and how to resource extra workload arising from an expanded purview.

Faden et al’s account also exchanges ‘protectionism’ as a central governance principle for ‘justice’. This entails consideration of the risks and benefits of planned data uses at the group, community or society level, rather than just individual-level risks (p226-7) [44]. This requires a detailed account of distributive and social justice in the context of genomic cancer data uses. The former, broadly speaking, is said to be the ‘distribution of all rights and responsibilities in society’ (p226) [44] and the latter is how to decide whether planned data uses will result in unfair burdens for certain parts of society [45]. In respect of cancer genomics, operationalizing such an account within governance is important in order to address widening socioeconomic equalities in subgroups such as breast and colorectal cancer, and the overall ‘glaring lack of studies vital to promoting health equity’ [21].

##### The separationist account

A separationist account entails more recognition of why conventional distinctions in research and clinical ethics might matter for governance. For example, judging whether the social value of the research activity might, over time, be at the expense of care outcomes is an important concern [41]. This phenomenon has been described in the setting of personalised health monitoring in adaptive ML where the learning is argued to happen for the sake of others, not individuals’ own clinical needs [41]. As noted in this description, part of the added challenge for a governance approach is to be able to understand the detailed learning objectives of adaptive ML given the opacity of such ML techniques [41]. Even so a presumption that ML in uses for clinical care is likely to have a ‘learning’ (research) element gives reason to enquire routinely about the non-clinical objectives of ML-driven data uses within governance.

Understanding the context within which data uses happen and findings are generated is also important in order to distinguish (and judge the ethics of) research and care aspects. While the genomic cancer case is not a direct correlate with the example of personalised health monitoring, clinical scientists, clinicians and patients also have to grapple with the fact that genomic findings cannot always be regarded as clinically significant. As technologies are applied in an agnostic setting (i.e. not driven by particular clinical phenotypes) more genomic variants will be found whose clinical significance is uncertain [46]. Deciding clinical significance therefore becomes not just a technical question of using variant interpretation guides and reference libraries well, but also relying on close links with research that can evaluate the significance of findings in different contexts, including different populations. Additionally, practical ethical questions accompany significance decisions, such as judging when and how to include variants on a clinical report, when to revise them, and how to re-contact patients over time [47]. This shows the importance at least to some stakeholders (patients, clinicians, scientists with clinical responsibilities) of distinguishing the different clinical versus research implications of uses. However, it also shows the inter-dependence of iterated, non-linear research-clinical processes within genomic data uses, and how, unlike the personalised health monitoring example, the aims and intention behind uses will be similarly inter-dependent, and often mutually beneficial.

This is one demonstration of how being too separationist in the governance approach will be unwieldy and will unhelpfully stifle learning from data uses. Additionally, as with most research proposals, one major value of novel use cases will be their exploratory nature, making it unclear how possible it is for applicants to governance to predict all relevant ethical considerations. Nevertheless, any predictions will help, progressively, to characterise the ethical landscape of use. This sees governance itself as a learning entity, a repository for institutional knowledge about what ethical considerations are pertinent to use, what frameworks to use, where to draw the line and make trade-offs etc.

Both hybrid and separationist accounts, then, are useful to consider for governance, the first to characterise the data and describe how practically uses will go, the second to highlight the practical and ethical implications for care which follow from data uses.

## Results: a PPIE role within a governance approach tailored to genomic data use in fields like cancer

What does all of this mean for the governance of genomic data uses in fields like cancer, and for PPIE’s role in that? Following Goldacre et al., we have argued that effective PPIE is a key factor in well-founded public trust and confidence in health data analysis and research [6]. We have argued that PPIE within the governance of big genomic data repositories or initiatives has the potential to foster legitimate grounding for public support. To this end we think that developing a coherent and effective PPIE role within the governance of cancer genomics is needed. In what follows we recommend a PPIE role within a governance approach which is sensitive to key ethical challenges of genomic data use in fields such as cancer and here set out a number of key governance aims^6^.

### Inclusive and representative

The PPIE role should be *inclusive* and *representative* of a cross section of publics or society. The latter might necessitate members holding vested interests—indeed this is to be expected and welcomed to an extent. This is because vested interests when well managed can allow members to, for example, represent and advocate for certain underserved groups or disease areas. However, members should be self-aware about how such interests may affect their approach to governance (e.g. a broad techno-optimism about science among patients contrasted with more caution among publics) and corresponding judgements, should be able to state this to others and have their judgements reasonably challenged as part of a procedurally justified decision-making process [48]. This helps raise awareness of the subtlety and complexity of COIs among members without more standard commercial- or research-based interests.

### Working in the public interest and transparency

The PPIE panel should also have ways to connect to and serve the interests of wider publics. This will firstly be part of their inclusion and representation work e.g. connecting to PPIE networks which look to minority group cancer interests [49]. Secondly, it will be in order to make governance *more transparent and accountable*, and so worthy of public support. This is in so far as PPIE members, through their governance work, can promote relevant and sufficient understanding among wider publics of information about how genomic data is governed and used in their care. The panel should especially comprise or connect to members of underserved communities, in order to gain better understanding of ethical issues pertaining to them, be they risks of harms to groups or other areas of inequality in data uses [50]. In this way, their role also helps social accountability within governance, since they are helping to involve ‘potential data subjects’ in governance considerations (p9) [32].

### Addressing inequity

Thirdly,—and also addressing an area of profound public interest—a PPIE role should address *concerns of inequity* in the collection and use of genomic data for cancer care. This should be in the context of governance which is sensitive to data aggregation and diversification attempts. Governance should also develop justice as a principle central to its deliberations in order to support the range of issues arising from the hybrid nature of data uses [42, 51]. This involves weighing the risks and benefits of genomic data uses at the population level and considering whether uses are just, particularly in light of any existing inequities in genomic cancer care or research metrics. It also means recognising that a just provision of cancer care through genomics is about adequate and fair contributions from groups to its societal aims, so balancing protections of groups with societal well-being. This balancing work is a complex part of the role and depends on developing and operationalising an account of distributive and social justice as applied to the case of genomic data uses in cancer, building on similar types of endeavour such as the LHS and research which considers the case for its ethical governance or related checks and balances [52, 53]. (It is envisaged this starts out as a set of principles which is refined by experience of being on the panel and considering different sets of uses and may also be informed by expert opinion, changes in social policy, etc.). Despite recognising the hybrid territory inhabited by genomic data uses, it is important to monitor whether data uses are pursuing generalizable knowledge at the expense of adequately serving the needs of individuals. This recognises the importance of separating research ethics and clinical ethics analyses in order to highlight the practical and ethical implications for care.

### Managing risk

The PPIE role may be multifaceted when it comes to questions of risk. As above, it could be valuable in discerning what planned uses might lead to risks, and whether these risks are justified by the aims of the project or projected compensating benefits. Secondly, the role could help to suggest what controls and security measures are possible to mitigate risks. This could be recommending use of a Trusted Research Environment [6, 54] or other secure environment as well as discussing feasible alternatives (e.g. if data projects cannot afford to use these options). Importantly, the emphasis should be on managing risks and burdens (as well as benefits) fairly as part of a justice-based account.

### Managing legacy through learning governance

Finally, PPIE panels can more effectively serve a governance approach if they are standing panels which meet regularly. This gives them an ongoing, incremental knowledge of the area of genomic data and cancer care and an opportunity to broaden their governance expertise and value to the process. It also avoids a sense of tokenism since they are integral to the governance process and are not used sporadically. This represents a form of iterative data governance where PPIE panels are authentically involved in the sense they can appreciate over time what their input is achieving, and their iterated contributions count towards the learning of the system. Thinking of governance itself as a learning system reflects its potential as a forum for a dynamic, multidirectional process of knowledge-making and -receiving with PPIE at its core [42, 55]. It may be that more than one panel is needed to manage workload, specialised uses, or uses which involve particular underserved groups. Panels should be appropriately trained prior to their involvement and remunerated especially if taking on longstanding governance commitments.

## Conclusion

Including PPIE as a key component of effective governance capable of fostering well-founded public support might help address barriers to the data uses within comprehensive cancer data collections. Such a role for PPIE fosters a more dynamic exchange where researchers, patients and the public contribute to collaborative environments for science and knowledge to flourish. Cultivating such environments is essential to ensure that research not only drives scientific progress but also aligns with societal needs and values. In this paper we have considered how PPIE should function within appropriate governance for genomic data for cancer care. We analysed two public attitudes studies about the role of PPIE in governance of genomics. This was set alongside analysis of two ethically-significant features of genomic research: data aggregation and novel group formation, and issues of hybridisation. This functioned to more fully determine a role for PPIE, situated in a governance approach sensitive and tailored to key areas of ethical consideration.

Our analysis led us to suggest a PPIE role with the following aims:

1. the role should include and represent a cross section of publics or society. Any vested interests of PPIE members (and other members) should be embraced so far as they contribute to a procedurally justified governance process.
2. the role should connect to and serve the interests of wider publics, also helping to promote a basic understanding of genomic data governance to aid public support.
3. it should be oriented to concerns of inequity in data uses, employing a governance framework where social justice plays a central role.
4. the role should help make judgements about risk and benefits, also being aware of appropriate risk mitigation controls.
5. the panel should operate as a standing panel which meets regularly (and is remunerated appropriately), broadening their knowledge, experience and value to the process, and making governance itself a learning system.

This is not to under-estimate the complexity or breadth of the governance approach we propose – it is likely that multiple bodies would be needed, administered by an overarching strategic administration, in order to manage increasing volumes and proliferating categories of use, even within the same data repository or registry. Additionally, further ethics research is needed to tackle complex questions around how to operationalise justice-based accounts of ethical governance effectively, given the proposal to move away from protectionism.

## Footnotes

1 Definitions and practices of Patient and Public Involvement and Engagement (PPIE) are highly variable and are accompanied by a rich history of patient advocacy and activism. For the purposes of this paper, we use the description of PPIE in reference [6] as a working definition: ‘The most useful, successful, and impactful health data research projects are often those that: design projects with, and for, patients and the public from the outset; involve a diverse range of representatives in every decision, from data definitions, to interpretation and dissemination; listen to (and act on) the advice, feedback, and input of patient representatives; and treat their values, beliefs and experiences as crucial to success…’ (6, p145). For more information about the origins and history of PPIE in England see [7], in Europe see [8], in the US see [9].

2 A declaration that Michael Parker was a co-author of [14].

3 The report defines the social contract as follows: ‘where patients, the wider public, health professionals, and researchers have shared expectations – a “social contract” – about what constitutes reasonable and acceptable uses of patient data and samples’.

4 ‘It’s impossible to know everything. Some degree of ignorance is inevitable, and we all must personally decide what is worth knowing and understanding. Rational ignorance helps each of us decide what information would be most useful. Rational ignorance means intentionally choosing to remain uninformed on a topic because the cost of acquiring the information is greater than the estimated potential benefits.’

5 ‘The notion of genetic citizenship refers to the obligations, rights, duties, and forms of care that circulate between citizens and the state’ p.38 [35].

6 We see the PPIE as central to decision-making within governance but also envisage other non-lay deciders within the governance approach.
